# Towards an Absolute Chronology for the Aegean Iron Age: New Radiocarbon Dates from Lefkandi, Kalapodi and Corinth

**DOI:** 10.1371/journal.pone.0083117

**Published:** 2013-12-26

**Authors:** Michael B. Toffolo, Alexander Fantalkin, Irene S. Lemos, Rainer C. S. Felsch, Wolf-Dietrich Niemeier, Guy D. R. Sanders, Israel Finkelstein, Elisabetta Boaretto

**Affiliations:** 1 Kimmel Center for Archaeological Science, Weizmann Institute of Science, Rehovot, Israel; 2 Sonia and Marco Nadler Institute of Archaeology, Tel Aviv University, Tel Aviv, Israel; 3 Merton College, University of Oxford, Oxford, United Kingdom; 4 Haferkamp 3c, Isernhagen, Germany; 5 German Archaeological Institute at Athens, Athens, Greece; 6 American School of Classical Studies at Athens, Corinth Excavations, Ancient Corinth, Greece; 7 Weizmann Institute-Max Planck Center for Integrative Archaeology, D-REAMS Radiocarbon Laboratory, Weizmann Institute of Science, Rehovot, Israel; Université de Poitiers, France

## Abstract

The *relative* chronology of the Aegean Iron Age is robust. It is based on minute stylistic changes in the Submycenaean, Protogeometric and Geometric styles and their sub-phases. Yet, the *absolute* chronology of the time-span between the final stages of Late Helladic IIIC in the late second millennium BCE and the archaic colonization of Italy and Sicily toward the end of the 8^th^ century BCE lacks archaeological contexts that can be *directly* related to events carrying absolute dates mentioned in Egyptian/Near Eastern historical sources, or to well-dated Egyptian/Near Eastern rulers. The small number of radiocarbon dates available for this time span is not sufficient to establish an absolute chronological sequence. Here we present a new set of short-lived radiocarbon dates from the sites of Lefkandi, Kalapodi and Corinth in Greece. We focus on the crucial transition from the Submycenaean to the Protogeometric periods. This transition is placed in the late 11^th^ century BCE according to the Conventional Aegean Chronology and in the late 12^th^ century BCE according to the High Aegean Chronology. Our results place it in the second half of the 11^th^ century BCE.

## Introduction

The absolute chronology of the different phases of the Iron Age in the Aegean has been debated during the last decade [1]–[9]. This is not surprising since any significant change in the dates would affect the entire Mediterranean basin, far beyond the Greek shores.

The disagreement regarding the chronology of the Aegean Iron Age is a result of an inherent problem in the archaeology of this region. The strength of the Greek painted pottery is in its robust *relative* sequence, including rapid stylistic changes. Yet, it is difficult to tie this relative scheme into an *absolute* dating system. This is so because the period between the final stages of the Late Helladic IIIC (LH IIIC) in the late second millennium BCE and the archaic colonization of Italy and Sicily toward the end of the 8^th^ century BCE lacks archaeological contexts that can be *directly* related to events carrying absolute dates, such as layers with Egyptian items bearing names of well-dated pharaohs. Scholars of the Aegean Iron Age who tried to resolve the problem have therefore been forced to resort to comparative material from the East, that is, to Levantine sites which yielded Greek Protogeometric (PG) and Geometric (G) items, such as the old excavations at Samaria, Megiddo and Tell Abu Hawam [10]. Yet, this too did not save the day, because: a) the Aegean items found in these sites did not come from stratigraphically secure contexts; b) the date of the relevant layers in the Levant was also debated [11], [12], mainly because it was founded on biblical texts whose historicity has been challenged [13]–[16].

**Figure 1:**
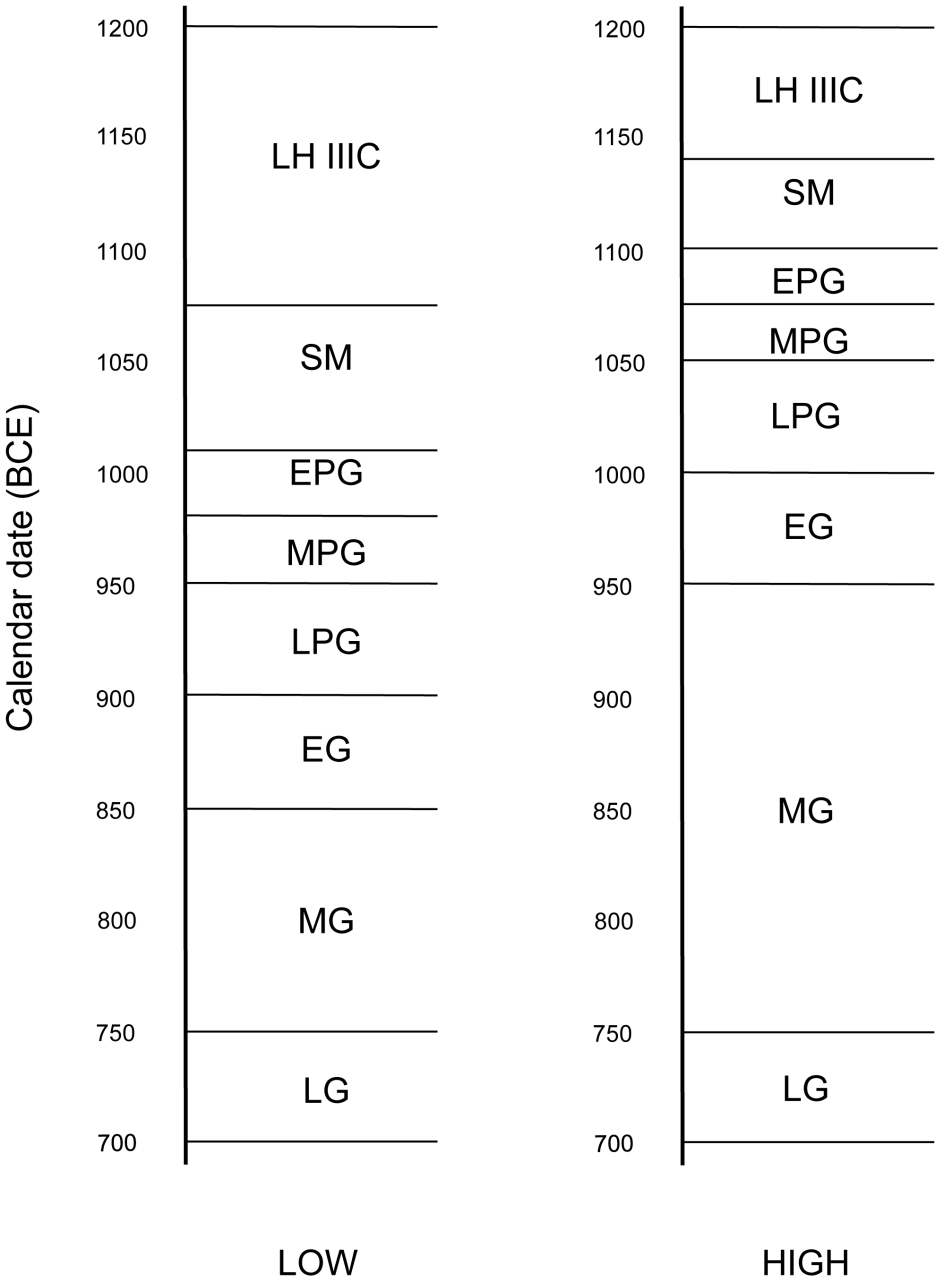
High and Low absolute chronologies for the ceramic phases of the Aegean Iron Age, based on [12], [17]. LH IIIC: Late Helladic IIIC; SM: Sub-Mycenaean; EPG: Early Proto-Geometric; MPG: Middle Proto-Geometric; LPG: Late Proto-Geometric; EG: Early Geometric; MG: Middle Geometric; LG: Late Geometric.

This resulted in two contrasting systems for the PG and G phases in the Greek world: the Conventional Aegean Chronology, which followed the Samaria-based Low Palestinian Chronology of Crowfoot-Kenyon, and the Aegean High Chronology based on the traditional High (biblical-based) Palestinian Chronology [17]. The appearance of the new Low Chronology for the Levant [14], [18], [19], which roughly corresponds to the old Low Palestinian Chronology of Crowfoot-Kenyon and therefore to the Conventional Aegean Chronology, has been embraced by the scholars working in the Aegean [12]. However, the debate is far from being resolved, because of recent attempts, based on radiometric dates from Italy, Spain and Tunisia [4], to raise the Aegean Chronology by more than a century. This would be in line with the High Palestinian Chronology, although the latter appears obsolete even according to the Modified Conventional Chronology for the Levant proposed by Mazar [20], [21], which differs from the Low Chronology of Levant by only a few decades. The renewed High Chronology for the Aegean has been criticized regarding unreliable contexts and problematic interpretation of the data [7], and the dispute continues [6] (Fig. 1).

The debate in the Levant involved approximately the same time-frame as in the Aegean – between the collapse of Egyptian rule in Canaan in the late 12^th^ century and the beginning of Assyrian domination in the late 8^th^ century [14]. In order to resolve it, starting in the late 1990s scholars turned to radiocarbon dating, with impressive success [19], [22], [23]. Though the results have been interpreted in different and sometimes contrasting ways, a general trend toward the Low Chronology is well-established by now [18], [20]. Dates for some Aegean Iron Age sites, mostly peripheral, have also been published [1], [2], [24]–[27], but so far a *systematic* attempt to date the PG and G sequence in Greece by means of radiocarbon analyses has not been attempted.

**Figure 2:**
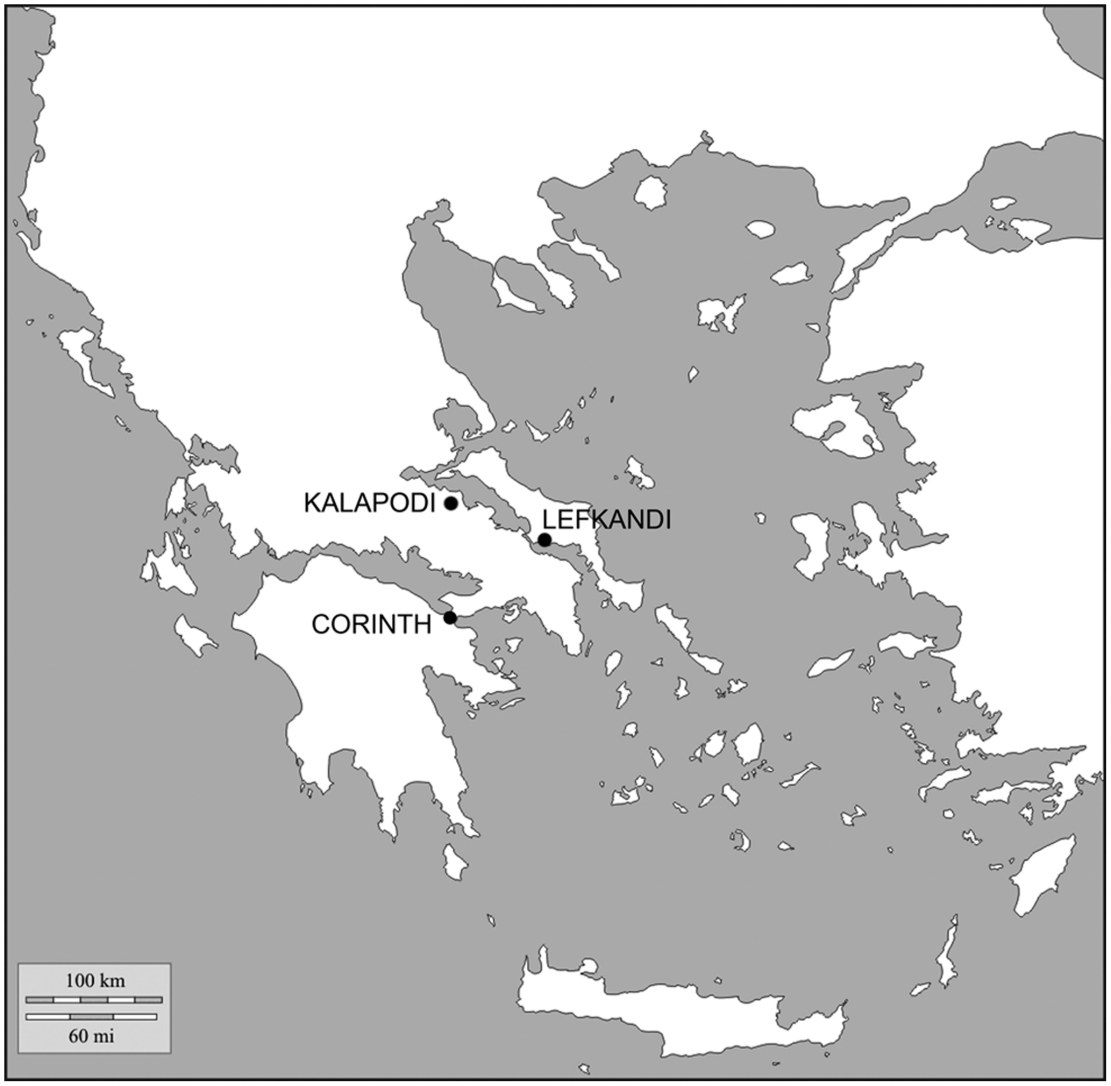
Map of the Aegean showing the locations of the sites mentioned in the article.

Establishing a reliable radiocarbon-based chronological system for the Iron Age phases in the Aegean depends on: a) secure stratigraphic contexts; b) with good pottery assemblages (which provide the basis for the *relative* Greek system); c) which can be dated by short-lived samples. This has been the goal of the radiocarbon track-team working in the framework of a European Research Council-funded project on Iron Age Levantine archaeology and the exact and life sciences. Here we report the results of our work in the last four years in three sites in Greece: Lefkandi [28], Kalapodi [29], [30], and Corinth [31] (Fig. 2).

## Materials and Methods

### Selection of sites and focus of research

The three sites discussed here were conceived as suitable for our purpose for a number of reasons:

1. They feature a stratigraphic sequence that covers a long time range from the LH IIIC through to the G period; the possibility of dating a full sequence is a key factor in the establishment of a solid absolute chronology, because this is the only way to determine transitions between phases and their duration using Bayesian analysis [32], [33].
2. They are located in the central part of Greece and represent the relative chronological sequence of three important regions, namely Euboea (Lefkandi), Phocis (Kalapodi) and Corinthia (Corinth). The links between the ceramic assemblages of these areas are well known, as well as those with neighboring regions, thus allowing a good control over the accuracy of the radiocarbon measurements vis-à-vis the relative system of the Aegean PG and G periods.
3. They have been carefully excavated and studied for decades and the given expeditions are still operating in the field, a situation that facilitated the retrieval of samples from museums, storage facilities and the field, and provided access to the relevant documentation.

The characteristics mentioned above are essential in order to obtain a reliable set of radiocarbon dates, but they are not enough. Samples should come from primary contexts which did not undergo any type of disturbance after their deposition, such as hearths and cooking installations, occupational accumulations on floors and single-burial inhumations. These contexts should feature well-defined ceramic assemblages, which provide the basis for the entire discussion. Destruction layers are especially important in this sense, as they represent short events in time and usually seal assemblages of complete vessels and clusters of short-lived samples, such as charred seeds, which are less likely to bear the “old wood” effect.

**Figure 3:**
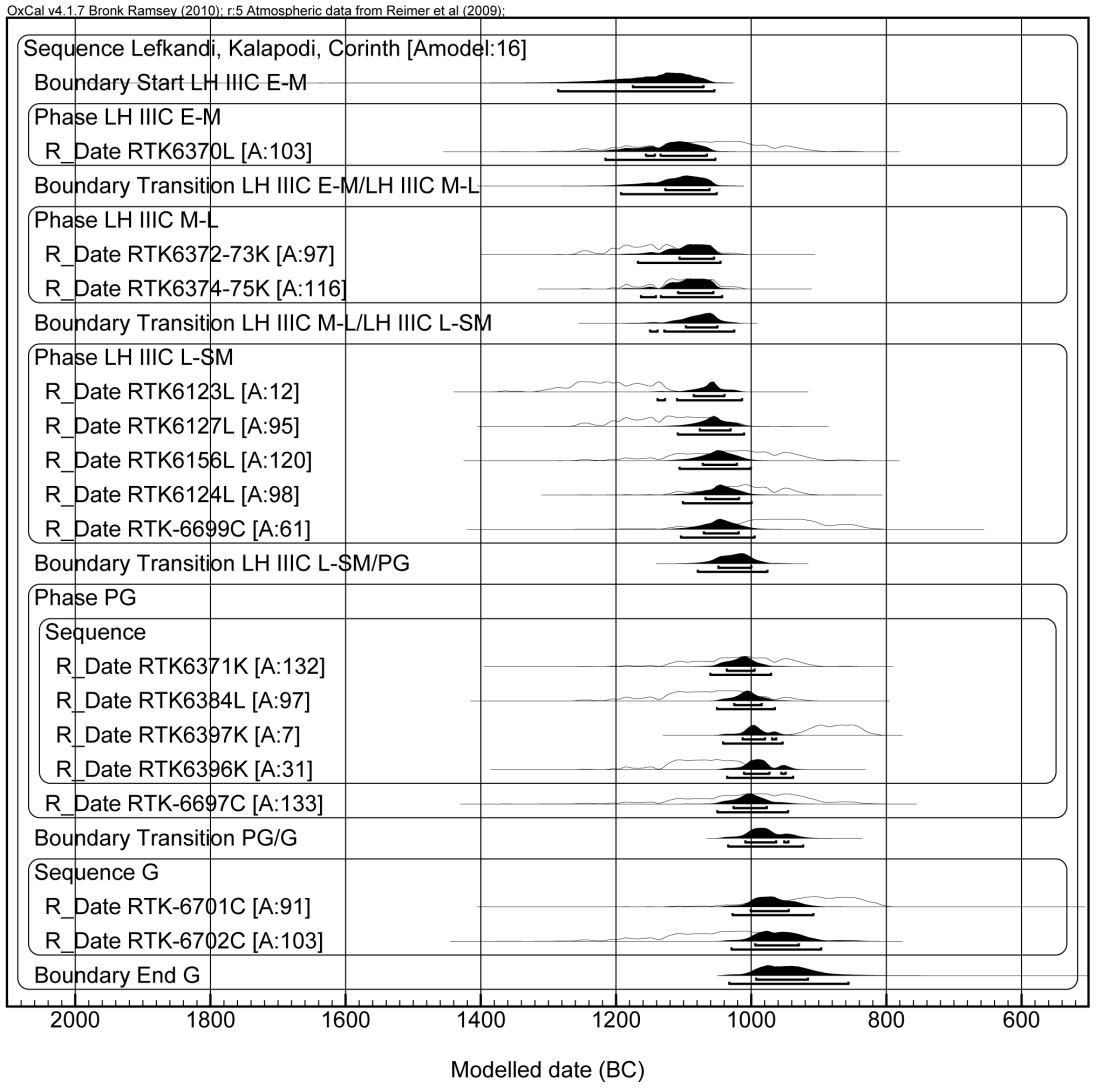
Model A, including all the dates. The label “L” after the laboratory number stands for Lefkandi; similarly, “K” stands for Kalapodi and “C” marks the samples from Corinth. Note that RTK-6372-73K, RTK-6374-75K, RTK-6123L, RTK-6124L, RTK-6127L, RTK-6371K, RTK-6384L, RTK-6397K and RTK-6396K are radiocarbon dates obtained by averaging multiple measurements of the same sample using the R_Combine command of OxCal.

**Figure 4:**
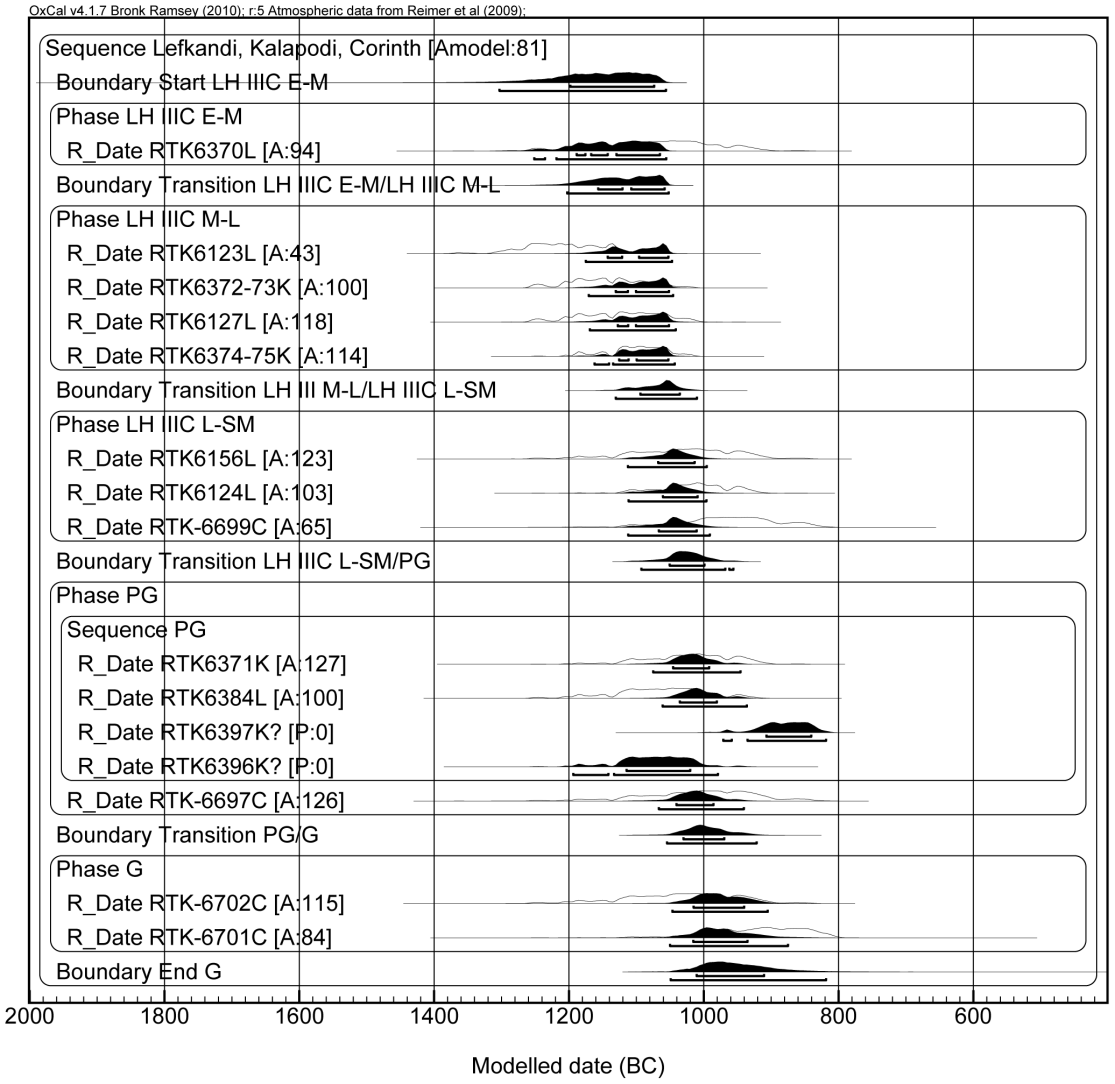
Model B. Note that two dates were labeled as outliers (RTK-6396 and RTK-6397), whereas RTK-6123 and RTK-6127 where placed within the LH IIIC Middle to Late phase.

**Figure 5:**
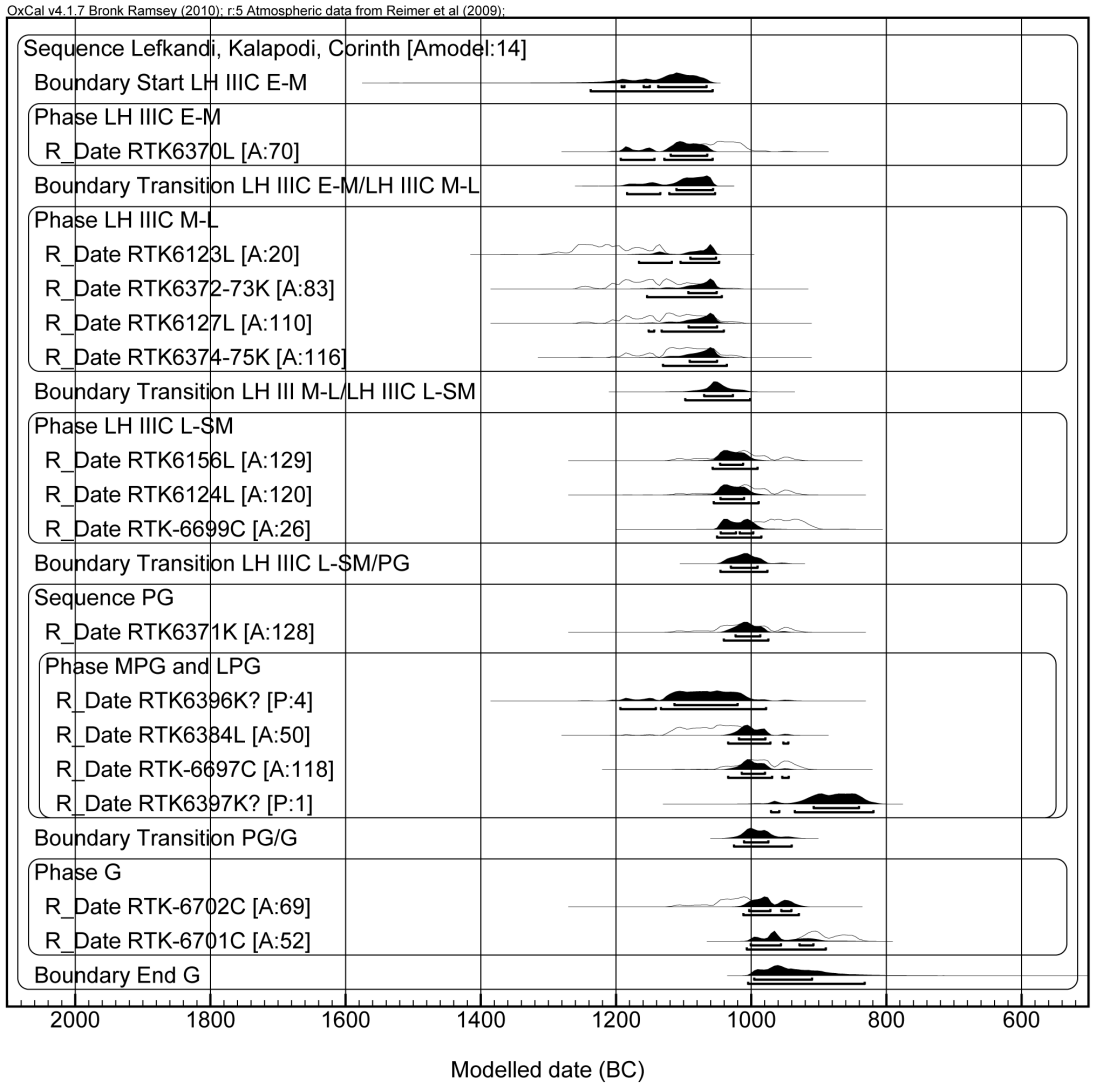
Model C. Note that the standard deviation of all the dates was reduced to ±20 yr BP.

**Figure 6:**
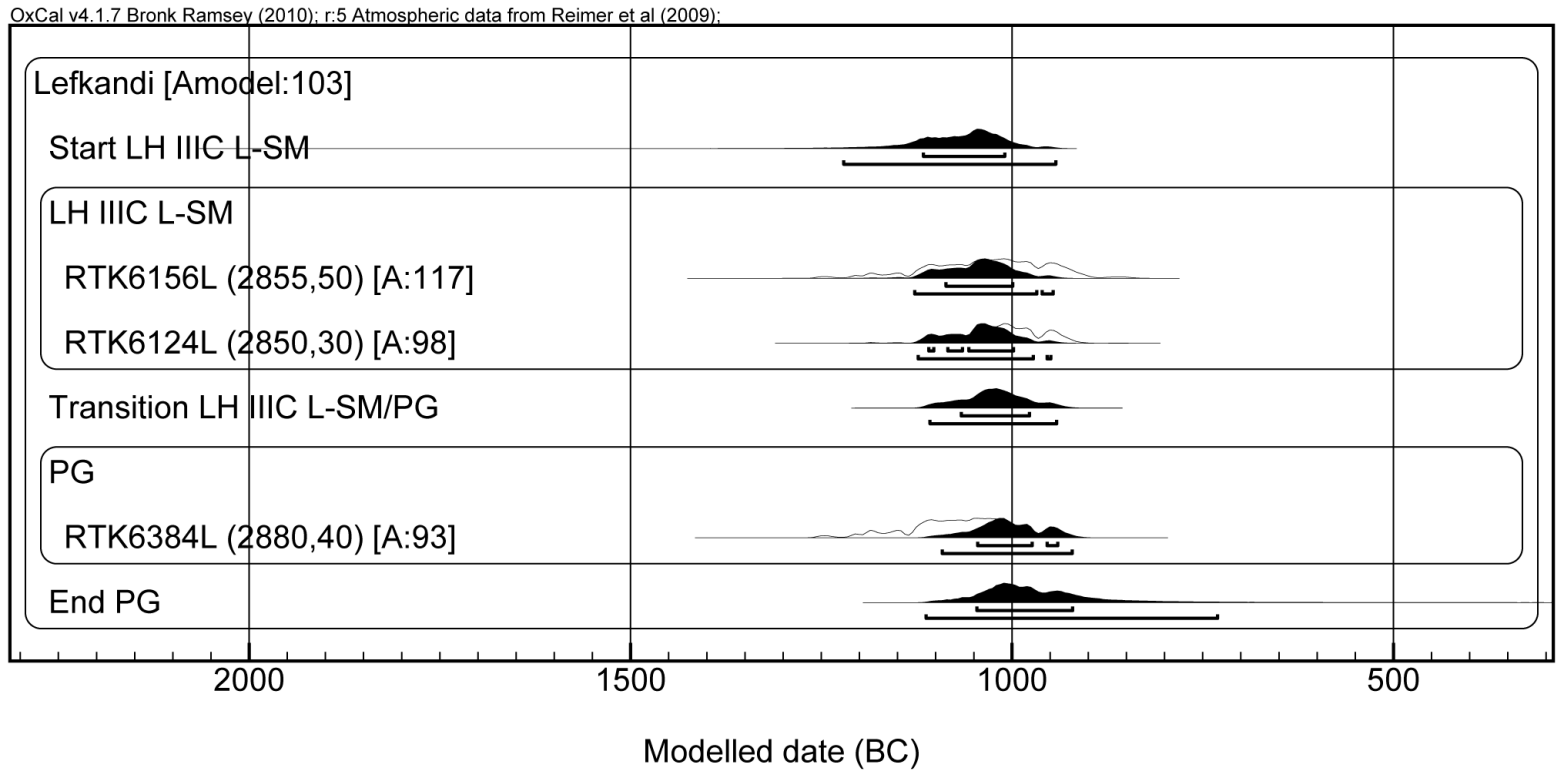
Model D. The plot shows the radiocarbon dates of Lefkandi only.

Some of these “ideal” criteria could not be applied in the sites under investigation. First, no destruction layers were found (they are not easy to come by in Greek sites), and therefore we were not able to collect samples from contexts with rich assemblages of complete vessels (e.g. Lefkandi and Kalapodi). As a result, in most cases we did not work with clusters of short-lived samples, such as olive pits and grain seeds. Also, when a good pottery assemblage was available, in several cases we could not find preserved collagen in bones. This is so for the horse bone from the famous Toumba in Lefkandi [28] and for some of the graves that were tested in Corinth [34]–[36] (results not included in this study). Still, we were able to collect a substantial amount of material, representing the LH IIIC, Submycenaean (SM) and PG periods (16 samples; Table 1). Evidently, they are not enough for a comprehensive dating of all the phases of the Iron Age. For instance, the PG/G transition could not be determined with precision, as only three dates (in addition to the 16 mentioned above) are available for the G period. Yet, what we did obtain allows the calculation of the highly important SM/PG transition using Bayesian analysis [32], [33] (this transition is labeled as LH IIIC Late-SM/EPG in the Bayesian models, Fig. 3–6). Good results for this transition can serve as a crucial peg for the Aegean Iron Age chronology. This is the focus of our article.

**Table 1:** ^14^C results obtained for the samples from Kalapodi, Lefkandi and Corinth with ceramic phase, laboratory number, material identification, context description, sample ID, ^14^C age in yr BP, calibrated ranges for ±1σ and ±2σ, and ‰ δ^13^C.

Ceramic phase	Lab#	Type	Context	Site and sample ID	^14^C age ±yr BP	Calibrated range ±1σ (BCE)	Calibrated range ±2σ (BCE)	δ^13^C (‰)
LH IIIC early to middle	RTK-6370	Charred wheat	Pit	Lefkandi, Basket 879, Area T North	2880±55	1187-1183 (1.1%), 1154-1146 (2.0%), 1130-976 (63.9%), 952-947 (1.2%)	1258-1233 (2.9%), 1217-916 (92.5%)	−22.7
LH IIIC middle to late	RTT-6104	Charcoal	Layer of burnt debris in standing section	Lefkandi, Area M, section 367	2980±55	1305-1126 (68.2%)	1388-1047 (95.4%)	−23.3
LH IIIC middle to late	RTK-6372 and RTK 6373	Charred wheat and chickpeas	Burnt debris	Kalapodi, KAL08 363 5035/4975	2930±25* ^,^ a (6)	1208-1202 (2.6%), 1195-1140 (34.3%), 1135-1111 (14.5%), 1103-1076 (12.6%), 1065-1056 (4.2%)	1259-1232 (6.0%), 1218-1041 (89.4%)	−23.4 (RTK6372), −22.4 (RTK6373)
LH IIIC middle to late	RTK-6374 and RTK 6375	Charred chickpeas and wheat	Hearth	Kalapodi, KAL08 368 5035/4975	2913±17* ^,^ b (8)	1129-1051 (68.2%)	1195-1142 (18.4%), 1134-1021 (77.0%)	−22.8 (RTK6374), −23.8 (RTK6375)
LH IIIC late to SM	RTK-6123	Animal bone	Pit	Lefkandi, Area M, Pit 47, basket 5359	2975±30* (4)	1263-1190 (47.7%), 1180-1158 (11.9%), 1145-1131 (8.5%)	1369-1359 (0.7%), 1315-1113 (93.7%), 1099-1088 (0.8%), 1063-1059 (0.3%)	−19.0
LH IIIC late to SM	RTK-6124	Animal bone	Pit	Lefkandi, Area M, Pit 48, basket 5366 (top)	2850±30* (4)	1052-973 (57.7%), 958-939 (10.5%)	1116-925 (95.4%)	−19.4
LH IIIC late to SM	RTK-6127	Animal bone	Pit	Lefkandi, Area M, Pit 48, basket 5374 (bottom)	2920±30* (4)	1193-1174 (9.5%), 1166-1143 (11.6%), 1133-1051 (47.1%)	1256-1237 (3.7%), 1215-1014 (91.7%)	−19.6
LH IIIC late to SM	RTK-6156	Animal bone	Pit	Lefkandi, Area M, Pit 47, basket 5359	2855±50	1114-1097 (6.0%), 1092-972 (53.1%), 959-936 (9.1%)	1209-901 (95.4%)	−20.4
SM	RTK-6699	Human bone	Single burial [32]–[34]	Corinth, COR1969-33	2805±55	1042-897 (68.2%)	1118-833 (95.4%)	−19.7
EPG	RTK-6371	Charred wheat	Burnt debris, possibly of a hearth	Kalapodi, KAL08 322 5035/4975	2850±40* (2)	1109-1104 (1.8%), 1072-1066 (2.3%), 1056-970 (50.3%), 962-932 (13.8%)	1187-1184 (0.3%), 1153-1149 (0.3%), 1130-906 (94.8%)	−22.5
(E?)PG	RTK-6697	Human bone	Single burial [32]–[34]	Corinth, COR1973-4	2835±55	1110-1104 (1.8%), 1074-1065 (2.5%), 1056-913 (63.9%)	1192-1176 (1.4%), 1163-1143 (1.7%), 1132-843 (92.3%)	−19.5
(E?)PG	RTK-6698	Human bone	Single burial [32]–[34]	Corinth, COR1973-5	2520±60	790-731 (19.9%), 692-660 (11.2%), 651-544 (37.2%)	800-486 (90.4%), 463-448 (1.7%), 443-416 (3.3%)	nd
MPG to LPG	RTK-6384	Textile	Toumba [28]	Lefkandi, LEFT9	2880±40* (2)	1126-1001 (68.2%)	1209-969 (89.8%), 963-930 (5.6%)	−26.0
MPG to LPG	RTK-6385	Decayed wood	Toumba [28]	Lefkandi, LEFT10	2600±55	833-753 (55.3%), 686-668 (7.5%), 632-626 (1.3%), 612-596 (4.2%)	896-731 (65.5%), 692-660 (9.5%), 651-544 (20.3%)	−24.6
LPG	RTK-6397	Animal bone	Fill with ceramics	Kalapodi, KAL05 146 5030/4965	2742±29* (4)	908-841 (68.2%)	972-959 (3.0%), 936-819 (92.4%)	−22.4
SPG	RTK 6396	Animal bone	Sacrifice place related to altar	Kalapodi, KAL05 131 5030/4965-70	2888±28* (4)	1116-1021 (68.2%)	1194-1142 (7.9%), 1133-979 (87.5%)	−19.7
(E?)G	RTK-6701	Human bone	Single burial [32]–[34]	Corinth, COR1969-29	2760±60	976-952 (10.4%), 946-833 (57.8%)	1049-805 (95.4%)	−19.4
MG	RTK-6702	Human bone	Single burial [32]–[34]	Corinth, COR1971-2	2855±55	1115-971 (58.7%), 960-935 (9.5%)	1252-1241 (0.7%), 1213-897 (94.7%)	−19.6
MG to LG	RTK-6395	Animal bone	Fill with ceramics	Kalapodi, KAL05 70 5030/4965	2445±55	746-689 (18.3%), 664-646 (5.7%), 552-412 (44.2%)	761-682 (22.3%), 671-405 (73.1%)	−19.2

The samples are ordered according to the relative chronological sequence. LH IIIC: Late Helladic IIIC; SM: Sub-Mycenaean; EPG: Early Proto-Geometric; MPG: Middle Proto-Geometric; LPG: Late Proto-Geometric; SPG: Sub-Proto-Geometric; EG: Early Geometric; MG: Middle Geometric; LG: Late Geometric;

* : the date is the average of multiple measurements on the same sample (the number of measurements is indicated between brackets);

a : RTK6372 and RTK6373 are parts of the same assemblage of seeds, and therefore dates were averaged;

b : RTK6374 and RTK6375 are parts of the same assemblage of seeds, and therefore dates were averaged.

All the charred seeds come from clusters. References for published contexts are included within the “Context” column; all the other contexts are yet unpublished. δ^13^C values were obtained using an isotope ratio mass spectrometer.

### Sampling and measurement strategy

The original idea was to collect samples from the field, but the strict quality-control criteria employed in screening the archaeological contexts [4], [19], [37], [38] left no suitable items. Nevertheless, it was possible to choose samples collected during past excavation seasons. As shown in [39], it is inappropriate to average dates obtained from different single short-lived samples even if they are found within the same layer and sometime even specific context; since the relationship between single samples is often unknown, they might have been deposited in different events in the history of a given layer, which can be decades apart. Therefore, we opted for clusters of seeds and/or bones where possible. Also, we decided to measure more than once all the samples for which we had enough material, in order to increase the precision of the dates. We believe that this is an essential step when dealing with many time-spans such as the phases of the Aegean Iron Age, which could have been shorter than a typical AMS uncertainty in the measurement (±50 years). In one case – the Toumba of Lefkandi – we decided to sample long-lived material (wood), taking into account the paucity of short-lived samples within this context, which we consider “sealed” and hence very reliable. This date will be considered as a *terminus post quem*.

Samples were thus collected in 2009, 2010 and 2012 from the excavations' storages and from museums, and their provenience was carefully checked with the excavators. Samples were taken in cooperation with the directors of the excavations (among the authors) and with the necessary permits from the Greek Ministry of Culture. Not working in the field, it was impossible to analyze the sediments associated with the samples in order to get more information about site-formation and post-depositional processes which could have affected the contexts [38], [40]. Only the context of sample RTT-6104 was analyzed using the microarchaeological approach of [39]. The sediment associated with this charcoal sample turned out to be in secondary deposition (see below), and therefore not reliable from a stratigraphic point of view. Nevertheless, it was dated in order to get an idea of the degree of “noise” from residual material within the stratigraphic/ceramic sequence. Bone samples were pre-screened at the sampling location in Greece in order to have a first glimpse regarding the preservation of collagen. In the case of Corinth, for instance, 13 samples were pre-screened, but only 7 yielded an insoluble fraction. About 200 mg of bone powder (ca. a full teaspoon) obtained by manually grinding clean bone fragments with an agate mortar and pestle were dissolved with ∼20 ml of 1N HCl inside a plastic ziplock bag. The occurrence of an insoluble fraction, floating within the solution and resembling gel, was a good hint for the presence of collagen [40].

### Laboratory procedures

The selected bones were further analyzed by means of Fourier-Transform infrared spectrometry (FTIR) in the Radiocarbon Laboratory at the Weizmann Institute of Science [41]. A total of 19 samples (Table 1) were processed for radiocarbon dating. Charred seeds, charcoal, bones, wood and textile were pre-treated at the Weizmann Institute of Science following [41], [42] in order to remove all contaminants. The procedure for charred remains is based on the acid-base-acid (ABA) method as in [41], [42], whereas bones were further subjected to ultra-filtration as in [41]. After the pre-treatment, the degree of purity of the charcoal and collagen was determined using FTIR spectrometry. Then, the samples were oxidized in vacuum with CuO at 900°C and prepared as graphite for ^14^C determination using accelerator mass spectrometry (AMS). The amount of carbon obtained was enough (40% or more) for the AMS measurements. Measurements were performed at the NSF-AMS Radiocarbon Laboratory at University of Arizona, Tucson. Radiocarbon dates are reported in conventional ^14^C years before present (BP) following the international convention [43]. All calculated ^14^C ages have been corrected for isotopic fractionation based on the stable carbon isotope ratio (δ^13^C value). Calibrated ages in calendar years have been obtained from the calibration tables of [44] using OxCal v 4.1.7 [33], [45]. The same software was used for the Bayesian analysis of the radiocarbon dates [32], [33], [45]. Carbon and nitrogen isotopic values were obtained using a Thermo Scientific EA1112 analyzer linked to a DELTA V isotope ratio mass spectrometer.

### Bayesian analysis

Groups of radiocarbon dates can be analyzed using Bayesian statistics [32], [33], [45]. This methodology allows the identification of outliers and their rejection based on a number of constraints provided by stratigraphic and contextual information. As a result, absolute chronological sequences become more precise and accurate.

When possible, the samples were collected from contexts which feature a single Aegean ceramic phase assemblage. Sometimes the ceramic evidence was less than ideal, forcing the excavators to label the context as belonging to a longer period, encompassing more than one relative ceramic phase, for instance “LH IIIC Late *to* SM”. In such a case, in the Bayesian models we maintained the excavators' affiliation and classified the two phases as belonging to one block. This procedure allowed us to check constraints which originate from the pottery sequence of the given sample: according to this method, a date that shows poor agreement in, e.g., the LH IIIC Late to SM phase might fit the model better if placed in the LH IIIC Late only, that is, with the phase labeled in the model LH IIIC Middle to Late. Similarly, samples labeled by the excavators as “PG” or “G”, with no further subdivision to early, middle or late, were considered as contemporary with the entire block (e.g. sample RTK-6697 is contemporary to other samples belonging to the Early Protogeometric, EPG or to the Middle Protogeometric, MPG).

The results of the analyses presented here as “transition” and reported as a time range should be regarded as calculated probability distributions of the transition timing; they do not represent the actual (historical) length of the transition [33], [45].

## Results

### Radiocarbon dating

The ^14^C calibrated ages are presented in Table 1, with the dates ordered according to the relative chronological sequence determined for the contexts from which the samples were collected. All the dates that were averaged (i.e. the dates of the samples measured more than once, Table 1) passed the χ^2^ test. Table 2 shows the C and N isotopic values of the human bones from Corinth. The δ^13^C and δ^15^N values were determined in order to check whether the bones are affected by a marine reservoir age effect dependent on a marine fish diet. Marine reservoir age can increase the apparent age of humans/animals by up to 400 years BP [46], [47]. Values lower than ca. −19‰ and 10‰ for the δ^13^C and the δ^15^N, respectively, are not compatible with a diet based on marine fish [48]. Therefore, based on the data reported in Table 2, we conclude that the individuals from Corinth are not affected by a marine reservoir age effect. Even if one takes the δ^15^N value of RTK-6702, which is slightly above 10‰, as an indicator for a possible input of marine fish in the diet of that specific individual, the reservoir age effect would increase the age of the sample by less than 20 radiocarbon years [48], with no substantial effect on the resolution of the chronological sequence that we propose in the present study. It was not possible to measure the δ^15^N value for RTK-6698, since the size of the collagen sample was very small and priority was given to the ^14^C determination. However its radiocarbon age, which is much younger than what was expected according to the ceramic phase attribution, clearly excludes the possibility of a reservoir age effect. The archaeological contexts of the animal bones from Lefkandi and Kalapodi are not published yet, therefore we are not able to provide precise taxonomic classifications. However, all the bones belong to large terrestrial mammals, which are unlikely to be fed on marine fish. The δ^13^C values for the animal bones (Table 1) support this assumption. Moreover, Kalapodi is located inland, not on the coast.

**Table 2:** Isotopic values of human bones from Corinth.

Lab #	%C	%N	C/N	δ^13^C (‰)	δ^15^N (‰)
RTK6697	40.0	16.8	2.7	−19.5±0.2	9.5±0.3
RTK6698	42.6	nd	nd	nd	nd
RTK6699	42.0	17.5	2.8	−19.7±0.3	9.7±0.5
RTK6701	42.0	18.8	2.6	−19.4±0.3	8.1±0.2
RTK6702	40.7	19.8	2.4	−19.6±0.1	10.4±0.5

Four samples were not used in the Bayesian analysis. Sample RTT-6104 was found in secondary deposition. The charcoal fragments were embedded in an ashy layer, but no combustion feature was visible by naked eye. FTIR analysis confirmed this observation, as no heat-altered clay minerals were detected within the same sample [49]. We conclude that the ash (and therefore the charcoal) is the product of a fire-related activity which took place elsewhere.

Sample RTK-6385 is ∼200 yr BP younger than expected, and this is probably related to poor preservation state or to its contamination during storage. FTIR analysis showed that this material does not contain cellulose as it would be expected for wood; rather, the FTIR spectrum resembles charcoal [41]. It is unlikely that the wood was found out of context, as it came together with other items from the well-defined Toumba context in Lefkandi.

Samples RTK-6395 and RTK-6698 are of little importance, as their radiocarbon dates fall within the “Hallstatt plateau” and the probability distribution covers a wide time span. Remarkably, RTK-6698 shows a date which is very late compared to the expected age provided by the ceramic assemblage. This might be related to a problematic context characterization at the time of the excavation. These four samples are therefore irrelevant for the purposes of the following discussion and were not included in the Bayesian analysis. All the absolute ranges presented in the text refer to ±1σ (68.2% probability).

### Bayesian models

The information regarding the relative pottery phases summarized in Table 1 was used as a constraint in our Bayesian analysis (lines of codes for each model are provided in Figure S1). Note that “Modelled date (BC)” notation at the end of each model relates to calendar years BCE. The modeled age probability distributions are presented as black areas, whereas the measured un-modeled age probability distributions appear as empty areas. Under each modeled distribution the upper segment refers to 68.2% probability, whereas the lower segment refers to 95.4% probability. After modeling some samples showed low agreement within the model (below 60% the date is considered to be in poor agreement [33], [45]). These samples were checked again to detect possible archaeological or analytical errors to justify their exclusion from the model. In the absence of evident errors the samples were excluded from the model once at a time until the general agreement of the model was above 60%. Each of the samples excluded is pointed out and justified. Model A (Fig. 3) presents 15 dates organized in a sequence of contiguous phases (i.e. one phase starts as the previous one ends, and from the oldest period of the relative chronology to the youngest). Within the PG phase was included a sub-sequence with dates organized according to the relative sub-phasing, where RTK-6371 belongs to the EPG, RTK-6384 to the Middle-Late Protogeometric (M-LPG), RTK-6397 to the LPG and RTK-6396 to the Sub-Protogeometrci (SPG). The only undivided PG date – RTK-6697 – was considered as contemporary with the entire block; in other words, it might be contemporary with any of the other samples. The agreement of the model is low (16%), and this is due mainly to sample RTK-6123 (too old for the LH IIIC Late to SM phase) and sample RTK-6397 (too young to be placed before RTK-6396, which comes from a SPG horizon and shows an older date). The SM/PG transition is placed between 1050 and 1000 BCE.

In Model B (Fig. 4) we decided to test another possibility, by placing samples RTK-6123 and RTK-6127 of the LH IIIC Late to SM in the LH IIIC Middle to Late phase, as explained above; this is still in line with the relative chronology sequence. As mentioned previously for Model A, RTK-6396 (SPG) and RTK-6397 (LPG) provided dates in an opposite order compared to the relative chronology of the ceramic phases they belong to (RTK-6396 older than RTK-6397). This is the reason for their exclusion by the model (i.e. by OxCal) on the ground of poor agreement (i.e. <60%). Considering that each of these two samples was measured four times and that the combined dates passed the χ^2^ test, we believe that the problem is in their ceramic-phase attribution; therefore they were labeled as outliers in OxCal. Also note that RTK-6396 is too old to be included within the SPG (Table 1). After these changes, the agreement increased to 81% and the SM/PG transition occurs between 1050 and 1000 BCE, which is similar to the result of Model A. The transition between PG and G ranges between 1030 and 970 BCE, but this value has little meaning, considering that there are only two dates for the G period. Moreover, one of these two samples (RTK-6702) shows a radiocarbon date that could fit also into the SM period. This explains the high range for the PG/G transition. In order to have a better control on this transition, more dates for the LPG, Early Geometric (EG) and Middle Geometric (MG) are needed. This we hope to achieve in next phase of our research.

In Model C (Fig. 5) the standard deviation error of all the dates of Model B was arbitrarily reduced by us to ±20 yr BP in order to check whether an increased precision might enhance the agreement of the model. We obtained the opposite effect, because the first five dates fall within the Late Bronze Age plateau of the calibration curve (approximately between 1300 and 1150 BCE); no matter how small the uncertainty in the measurement, these dates will always show a wide probability distribution. Also, the dates which do not fall in the region of the plateau (from the LH IIIC Late onwards), cluster in a shorter period of time, with no overlap with the older dates (those located in the plateau), thus resulting in a low agreement of the model. Yet, even in this case the SM/PG transition is located at the end of the 11^th^ century (1030-990 BCE). Remarkably, this happens also if we consider only the dates from Lefkandi, as shown in Model D (Fig. 6). In this case the SM/PG transition occurs between 1065 and 980 BCE. Therefore, even taking into account one specific site for which we have enough dates to calculate the transition, the range of the transition would not change.

It is clear that all the models presented above point towards the same result, i.e. they place the SM/PG transition in the second half of the 11^th^ century, approximately centered on 1025 BCE.

## Discussion

Adherents of the Conventional Aegean Chronology put the SM/PG transition in the mid-to-late 11^th^ century BCE, while those who accept the High Chronology place it close to the end of the 12^th^ century BCE. Accepting the latter would force one to stretch the MG over two centuries at the expense of the EG, the entire PG, the SM and even part of the LH IIIC [12] (Fig. 1). Our results support the Conventional Aegean Chronology [17] and shed light on the duration of the SM phase in the Aegean sequence, a phase that is still being considered as somewhat elusive [27].

Despite a plea to abandon the term [50], the SM represents a definable chronological stage, at least in Attica, Boeotia and the Argolid, even if from an interregional perspective it may overlap with the final stages of the LH IIIC Late and the beginning of the EPG (e.g., the LH IIIC Late in the Argolid is contemporary with the beginning of the SM in Attica). Moreover, even if one follows Rutter [50] and argues that the style referred to as SM represents tombs' repertoire, chronologically parallel to the LH IIIC Late in settlement sites (see Lemos [51] against this notion), it is not necessarily crucial for the purpose of establishing the absolute chronology for this stylistic phenomenon. From the perspective of relative chronology, given the paucity of the SM material and the dense sequence of styles in the Late Bronze and early Iron Ages, the SM phase could not have been long-lived. It is generally allowed two generations, that is, some 50 years of existence [52], [53] at the most [27]. Lemos' suggestion [51], which allows it two generations of twenty-five years long, with an additional generation for the transition from SM to the EPG may be too generous, since it turns a relatively short-lived, local phenomenon, without much internal variety [12], [54], into one of the longest phases in Iron Age Greece. The same holds true for the more maximalist reconstruction of Ruppenstein [55], according to whom the SM period covers most of the 11^th^ century BCE.

The absolute chronology of the SM period is difficult to establish [27]. The upper anchor is not clear enough, due to the lack of genuine LH IIIC Late imports in well-dated strata in the Levant [56]. The lower anchor, i.e. the transition to the EPG, is no less problematic. Desborough [57] suggested that the Attic PG started at ca. 1025 BCE; later on, he hesitantly opted for a slightly earlier date of ca. 1050 BCE [58]–[61]. This date was in fact based on Gjerstad's calculations [62] regarding the presence of a Cypriot White Painted I bowl (contemporary to the EPG in Greece) in Stratum VIA at Megiddo in the Levant, dated by Albright, on the basis of biblical references, to ca. 1050-1000 BCE [5]. A large number of ^14^C determinations now puts the beginning of Megiddo VIA in the second half of the 11^th^ century and its end in the range 985-935 BCE [63]. Thus, it seems that even before one applies the radiocarbon dates reported here, the initial date of ca. 1050 BCE for the SM/PG transition is probably too high [5]. A date closer to ca. 1020/1000 BCE, which would fit better the Low Chronology in the Levant would be in line with Desborough's initial guess and Mountjoy and Hankey's lowering of the beginning of the Protogeometric period [51], [64]. No less important, it fits the dates reported here. The recent revival of the High Aegean Chronology, based on radiometric dates from sites such as Assiros, Carthage and Huelva [1]–[4], [6], apart from being based on problematic interpretations [5], [65] and dubious contexts [7], would push the SM/PG transition toward the end of the 12^th^ century BCE, a date which is far from the range reported here.

According to Weninger and Jung [65], the SM/EPG transition should be set around 1070/40 BCE. We prefer to consider this datum as reflecting the beginning of the SM phase [7]. Based on the radiocarbon measurements reported here and the considerations detailed above, we suggest to put its end—and the transition to the EPG—around 1020/1000 BCE.

## Conclusions

The absolute dates of the relative ceramic phases of the Iron Age in the Aegean Basin have been disputed for many decades. In this article we report the results of the first systematic attempt to radiocarbon date these phases with samples taken from the key sites of Lefkandi, Kalapodi and Corinth. The data at hand enable to tackle the important transition between the SM and PG periods. This transition is placed by adherents of the Conventional Aegean Chronology in the mid-to-late 11^th^ century and by supporters of the High Aegean Chronology in the late 12^th^ century BCE. Our results put it in the second half of the 11^th^ century BCE.

## Supporting Information

Figure S1 **Lines of codes for the Bayesian models.**
(TXT)
